# The IL-33-induced p38-/JNK1/2-TNFα axis is antagonized by activation of β-adrenergic-receptors in dendritic cells

**DOI:** 10.1038/s41598-020-65072-3

**Published:** 2020-05-18

**Authors:** Christiane Helbig, Franziska Weber, Nico Andreas, Thomas Herdegen, Matthias Gaestel, Thomas Kamradt, Sebastian Drube

**Affiliations:** 1 Institute of Immunology, Jena University Hospital, 07743 Jena, Germany; 2 Institute for Experimental and Clinical Pharmacology, Medical School Schleswig-Holstein, Campus Kiel, 24105 Kiel, Germany; 3 Institute for Cell Biochemistry, Medical School Hannover, Hannover, 30625 Germany

**Keywords:** Immunology, Cytokines

## Abstract

IL-33, an IL-1 cytokine superfamily member, induces the activation of the canonical NF-κB signaling, and of Mitogen Activated Protein Kinases (MAPKs). In dendritic cells (DCs) IL-33 induces the production of IL-6, IL-13 and TNFα. Thereby, the production of IL-6 depends on RelA whereas the production of IL-13 depends on the p38-MK2/3 signaling module. Here, we show that in addition to p65 and the p38-MK2/3 signaling module, JNK1/2 are essential for the IL-33-induced TNFα production. The central roles of JNK1/2 and p38 in DCs are underpinned by the fact that these two MAPK pathways are controlled by activated β-adrenergic receptors resulting in a selective regulation of the IL-33-induced TNFα response in DCs.

## Introduction

The alarmin IL-33 which is passively released upon necrosis from endothelial and epithelial cells^1^ is relevant for the pathogenesis of allergic reactions^2–4^ by activating mast cells and DCs which express the T1/ST2 (the IL-33R)^3–6^. Binding of IL-33 to the IL-33R, a TLR-Interleukine-1 Receptor (TIR) family member^7^, results in association of the IL-33/IL-33R complex with the IL-1R accessory protein (IL-1RAcP)^8,9^. Subsequently, the IL-33/IL-33R/IL-1RacP receptor complex mediates the MyD88-dependent activation of the TAK1-IKK2 signaling node which results in IκB degradation and thus NF-κB activation^7^. IL-33 also induces a TAK1-dependent activation of MAPK pathways, such as the p38-MAPK-activated protein kinases 2 and 3 (MK2/3) signaling module, ERK1/2 and JNK1/2^7,10,11^. In DCs^5^, mast cells^4,12^, NK-cells^13^ and innate lymphoid cells (ILC2)^14^, the IL-33-induced cytokine response essentially depends on the p38-MK2/3 signaling module. This results in the production of TNFα^5^, a cytokine essentially involved in the pathogenesis of allergic inflammation^15,16^ and, thereby an attractive target for treatment of allergic reactions^17–19^. However, the IL-33-induced signaling events resulting in the production of TNFα and its regulation in DCs is not completely understood. To characterize IL-33-induced signaling pathways in DCs, we used bone marrow-derived dendritic cells (BMDCs), which are an *in vitro* model equivalent to inflammatory DCs^20,21^.

We show that beside NF-κB and the p38-MK2/3-signaling module^5^, JNK1/2 are essential to mediate the IL-33-induced production of TNFα in BMDCs. Interestingly, adrenergic receptors, which are expressed on DCs^22–24^, antagonize the IL-33-induced activation of JNK1/2 and p38 resulting in a selective inhibition of the TNFα biosynthesis, but not of the IL-6 production. Together, our data demonstrate a central role of JNK1/2 in the induction and regulation of the IL-33-induced TNFα response in BMDCs.

## Results

### JNK1/2 are essential for the IL-33-induced production of TNFα in BMDCs

**Figure 1:**
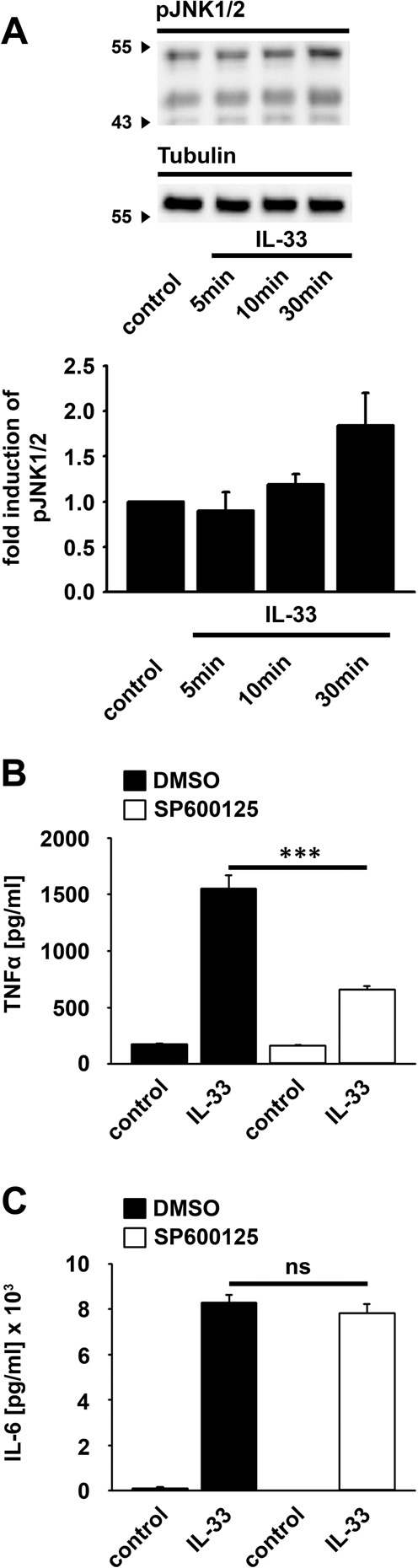
The IL-33-induced TNFα production depends on JNK1/2. **(A)** Wt BMDCs were stimulated with IL-33 (100 ng/ml) (as indicated). Lysates were analyzed by western blotting (n = 3). The original blots are shown in Supplementary Fig. 5. **(B,C)** Wt BMDCs were treated with SP600125 (5 µM). Afterwards cells were stimulated with IL-33 (100 ng/ml) (n = 3). Supernatants were collected and analyzed for TNFα **(B)** or IL-6 **(C)** (n = 3). Shown is the mean ± SD; ***p  <  0.001.

Splenic DCs do not express the IL-33R^2^. In contrast to this, GM-CSF-generated BMDCs express the IL-33R and are thus sensitive to IL-33 stimulation^5,25^. Therefore we used BMDCs as an *in vitro* model to investigate IL-33-induced signaling pathways in DCs. As recently shown in BMDCs^5^, IL-33 induces a MyD88-NF-κB-mediated TNFα production **(**Supplementary Fig. 1B–D**)** which also depends on the p38-MK2/3 signaling module **(**Supplementary Fig. 1E,F**)**. In addition, IL-33 activates JNK1/2 in BMDCs **(**Fig. 1A**)**. Inhibition of JNK1/2 by SP600125 reduced the production of TNFα **(**Fig. 1B**)** but not of IL-6 **(**Fig. 1C**)**. This demonstrates that beside the p38-MK2/3 signaling module^5^, JNK1/2 are essential for the IL-33-induced TNFα production, but are dispensable for the production of IL-6 in BMDCs. Due to the essential role of JNK1/2 and the p38-MK2/3 signaling module we focused our work on these MAPK pathways.

### JNK1/2 are dispensable for the IL-33-induced activation of IKK2 and p38

IL-33 induces a JNK-dependent TNFα response which also depends on IKKs and p38^5^. Therefore, we speculated that JNK1/2 activate IKK2 and/ or p38. However, neither the JNK1/2 inhibitor SP600125 **(**Supplementary Fig. 2A,B**)** nor JNK1 or JNK2 deficiency **(**Supplementary Fig. 2C–F**)** influenced the IL-33-induced activation of IKK2 and p38. Next we determined the influence of JNK1 or JNK2 in the TNFα and IL-6 production. As shown in Supplementary Fig. 2G neither JNK1 nor JNK2 deficiency reduced the IL-33-induced TNFα and IL-6 production in BMDCs. These data show that JNK1/2 are not involved in the IL-33-induced activation of IKK2 and p38 and that inactivation of all JNKs by SP600125, but not the specific inactivation of either JNK1 or JNK2, is prerequisite to reduce the production of TNFα.

### The p38-MK2/3 signaling module is dispensable for the IL-33-induced activation of IKK2 and JNK1/2

**Figure 2:**
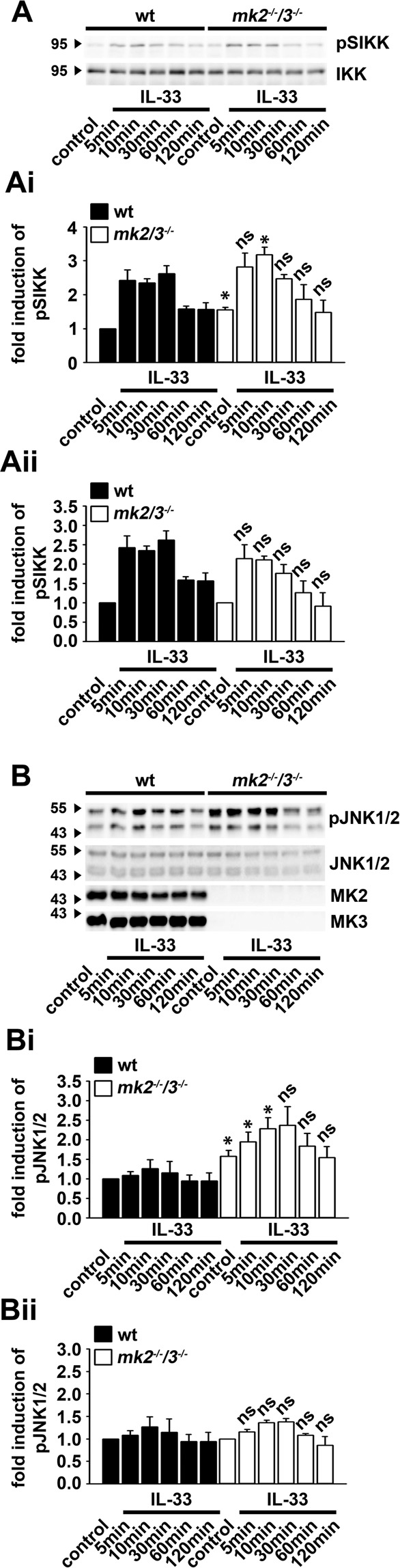
MK2/3 is not involved in the IL-33-induced JNK activation. **(A)** Wt and *mk2*^−/−^*/3*^−/−^ BMDCs were stimulated with IL-33 (100 ng/ml). Lysates were analyzed by Western blotting. Blots of 3 independent experiments with BMDCs separately generated from wt and *mk2*^−/−^*/3*^−/−^ mice were quantified and statistically analyzed. **(Ai)** The control of unstimulated wt BMDCs was set as 1 (shown is the mean ± SD from n = 3 independent experiments; ns: not significant; *p <  0.05). **(Aii)** The unstimulated controls of wt and *mk2*^−/−^*/3*^−/−^ BMDCs were set as 1 (shown is the mean ± SD from n = 3 independent experiments; ns). **(B)** Wt and *mk2*^−/−^*/3*^−/−^ BMDCs were stimulated with IL-33 (100 ng/ml). Lysates were analyzed by Western blotting. Blots of 3 independent experiments with BMDCs separately generated from wt and *mk2*^−/−^*/3*^−/−^ mice were quantified and statistically analyzed. **(Bi)** The control of the unstimulated wt BMDCs was set as 1 (shown is the mean ± SD from n = 3 independent experiments; ns: not significant; *p <  0.05). **(Bii)** The unstimulated controls of wt and *mk2*^−/−^*/3*^−/−^ BMDCs were set as 1 (shown is the mean ± SD from n = 3 independent experiments; ns: not). The original blots are shown in Supplementary Figs. 5 and 6.

JNKs together with the p38-MK2/3 signaling module mediate the production of TNFα upon IL-33 stimulation. P38 controls JNKs^26,27^ and the IKK complex^28–30^. We investigated whether the p38-MK2/3 signaling module mediates the activation of IKK2 and/ or JNK1/2 and thus the production of TNFα. Therefore, we used *mk2*^−/−^*/3*^−/−^ BMDCs. The basal activation (unstimulated control) of IKK2 and JNK1/2 **(**Fig. 2A,B and Ai,Bi**)** was increased in *mk2*^−/−^*/3*^−/−^ compared to wt BMDCs, and stimulation with IL-33 further increased the high basal activation of IKK2 and JNK1/2 **(**Fig. 2A,B and Ai,Bi**)**. To determine whether MK2/3 deficiency influences the IL-33-induced activation of IKK2 and JNK1/2, we calculated the fold induction of IKK2 and JNK1/2 in wt and *mk2*^−/−^*/3*^*−/−*^ BMDCs. Thereby, we set the unstimulated controls in wt and *mk2*^−/−^*/3*^−/−^ BMDCs as 1 and calculated the fold induction compared to the unstimulated controls in wt and *mk2*^−/−^*/3*^−/−^ BMDCs. As shown in Fig. 2Aii and Bii the fold induction of the IL-33-induced activation of IKK2 and JNK1/2 in *mk2*^−/−^*/3*^−/−^ was similar to wt BMDCs. This demonstrates that MK2/3 control the basal activity of IKK2 and JNK1/2, but not the IL-33-induced activation of IKK2 and JNK1/2.

Interestingly, IKK2 and JNK1/2 control mast cell proliferation^31^. We found an increased basal activity of IKK2 and JNK1/2 in BMDCs. Therefore, we hypothesized an elevated proliferation of *mk2*^−/−^*/3*^−/−^ compared to wt BMDCs. As shown in Supplementary Fig. 2H the basal proliferation rate of *mk2*^−/−^*/3*^−/−^ BMDCs was increased compared to wt BMDCs. These data demonstrate that the functional p38-MK2/3 signaling module is essential to control the basal activity of IKK2 and JNK1/2 as well as the proliferation of BMDCs. However, there are no crosstalks between the JNK1/2 and the p38-MK2/3 signaling module in response to IL-33 stimulation.

### IL-33 predominantly activates JNK2L in BMDCs

**Figure 3:**
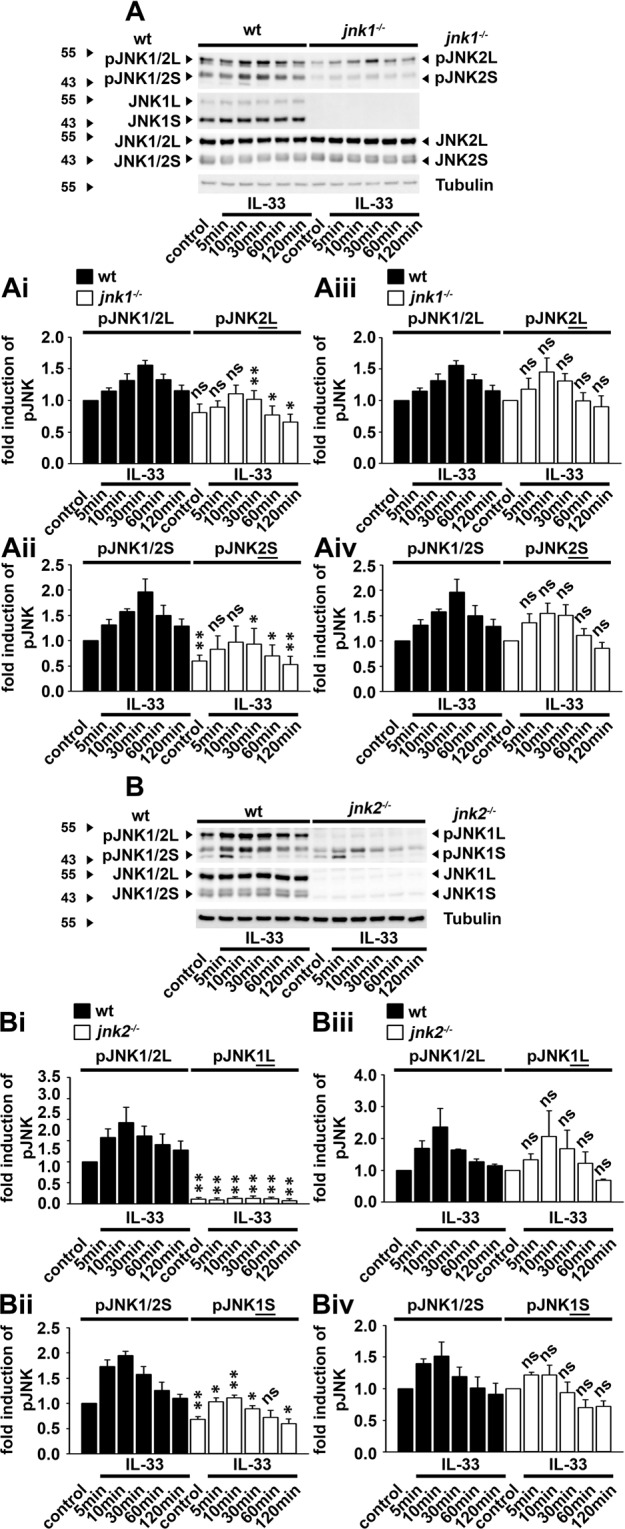
IL-33 predominantly activates JNK2L. **(A,B)** Wt, *jnk1*^−/−^ **(A)** and *jnk2*^−/−^ **(B)** BMDCs were stimulated with IL-33 (100 ng/ml). Lysates were analyzed by Western blotting. Blots of 5 (for wt/ *jnk1*^−/−^) and 4 (for wt/ *jnk2*^−/−^) independent experiments with BMDCs separately generated from wt, *jnk1*^−/−^ or *jnk2*^−/−^ mice were quantified, and statistically analyzed. **(Ai,Aii)** The control of the unstimulated wt BMDCs was set as 1 (shown is the mean ± SD independent experiments; ns: not significant; *p <  0.05 and **p  <  0.005). **(Aiii,Aiv)** The unstimulated controls of wt and *jnk1*^−/−^ BMDCs were set as 1 (shown is the mean ± SD independent experiments; ns: not significant). **(Bi,Bii)** The control of the unstimulated wt BMDCs was set as 1 (shown is the mean ± SD independent experiments; ns: not significant; *p <  0.05 and **p  <  0.005). **(Biii,Biv)** The unstimulated controls of wt and *jnk2*^−/−^ BMDCs were set as 1. (shown is the mean ± SD independent experiments; ns: not significant). The original blots are shown in Supplementary Figs. 7 and 8.

Next, we determined why neither JNK1 nor JNK2 deficiency influenced the IL-33-induced TNFα production. We hypothesized that a compensatory mechanism of the JNK isoforms in JNK deficient BMDCs. JNK1 and JNK2 are expressed as long (L) and short (S) isoforms (JNK1L/S and JNK2L/S)^32^. First, we evaluated the role of JNK1 isoforms by using *jnk1*^−/−^ BMDCs. Compared to the pJNK blots in wt BMDCs, the pJNK blots of the remaining JNK2L/S in *jnk1*^−/−^ BMDCs were reduced **(**Fig. 3A, Ai and Aii**)**. However, when the controls in wt or *jnk1*^−/−^ BMDCs were set as 1, the fold activation of the JNK isoforms in wt BMDCs was similar to the fold activation of remaining JNK2L and JNK2S isoforms in *jnk1*^−/−^ BMDCs **(**Fig. 3A, Aiii and Aiv**)**. This indicates, that inactivation of JNK1 reduced the total JNK activity in BMDCs without affecting the IL-33-induced activation of JNK2L/JNK2S in BMDCs. Next, we tested the role of JNK2 by using *jnk2*^−/−^ BMDCs. Compared to the pJNK blots in wt BMDCs, the activity of JNK1L was strongly reduced in *jnk2*^−/−^ BMDCs **(**Fig. 3B and Bi**)**. However, the activity of JNK1S in *jnk2*^−/−^ BMDCs was only slightly reduced compared to the pJNK blots in wt BMDCs **(**Fig. 3B and Bii**)**. This indicates that with the deletion of JNK2L in *jnk2*^−/−^ BMDCs, BMDCs lose the main JNK L isoform which contributes to the total activity of large JNK1/2 (pJNK1/2 L) isoforms. In contrast to this, with the loss of JNK2S in *jnk2*^−/−^ BMDCs, BMDCs lose the short JNK isoform which slightly contributes to the total activity of the small JNK1/2 (pJNK1/2 S) isoforms in wt BMDCs. However, the fold induction of JNK1L **(**Fig. 3B and Biii**)** and JNK1S **(**Fig. 3B and Biv**)** in *jnk2*^−/−^ BMDCs is similar to the pJNK blots in wt and BMDCs indicating that JNK2 deficiency also does not influence the IL-33-induced activation of JNK1S and JNK1L. The barely detectable activation of the remaining JNK1L isoform in JNK2 deficient BMDCs indicates that IL-33 predominantly induces the activation of JNK2L. However, the deletion of one JNK isoform is compensated by the remaining JNK isoform.

### Noradrenalin modulates the IL-33-induced cytokine production

**Figure 4:**
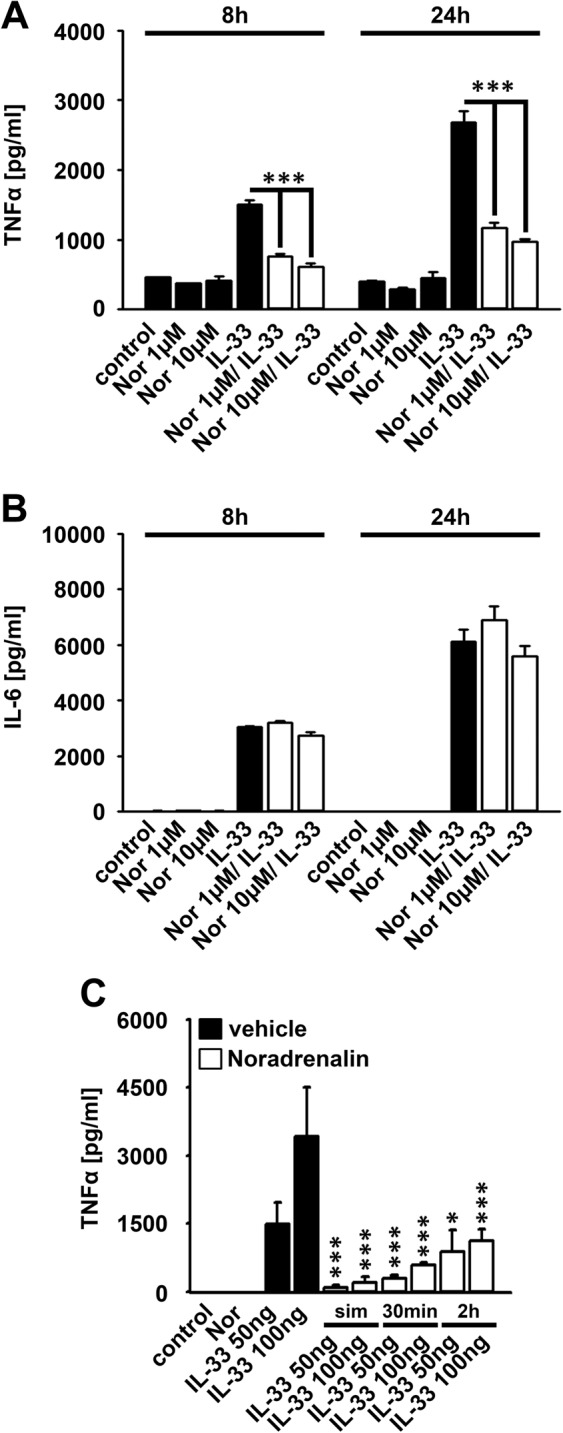
Noradrenalin inhibits the IL-33-induced TNFα production. **(A,B)** Wt BMDCs were either stimulated with Noradrenalin (as indicated) or IL-33 (100 ng/ml) (black columns) or both together (white columns). Supernatants were collected and analyzed by ELISA (shown is the mean ± SD; ***p  <  0.001) (n = 3). **(C)** Wt BMDCs were stimulated with either Noradrenalin (10 µM) or IL-33 (100 ng/ml) (black columns) or both together (white columns)  (sim: cells  were  stimulated  simultaneously  with  Nor and IL-33; 30min, 2h: cells were pre-stimulated with Nor for 30min or 2h prior to IL-33 stimulation). Supernatants were collected and analyzed by ELISA. (shown is the mean ± SD; *p <  0.05; ***p  <  0.001) (n = 3).

**Figure 5:**
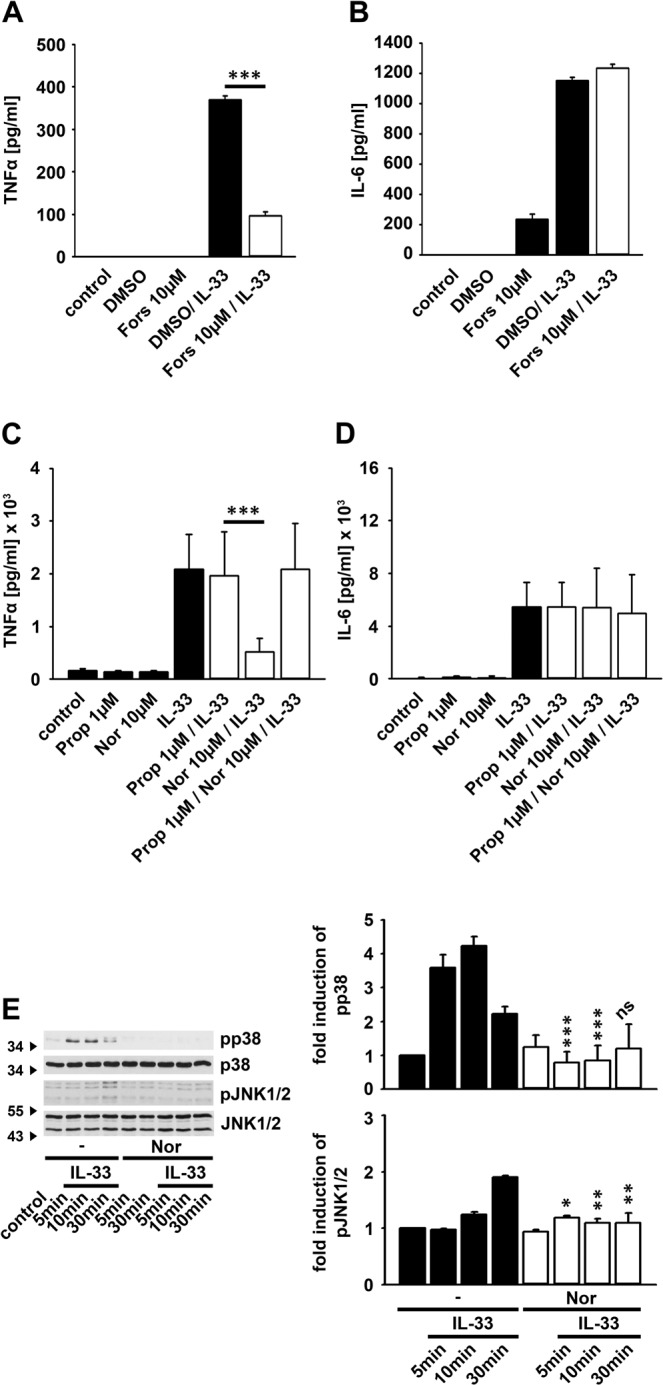
Forskolin inhibits the IL-33-induced TNFα production. **(A,B)** Wt BMDCs were treated with Forskolin (10 µM) and stimulated with IL-33 (100 ng/ml). Supernatants were collected and analyzed by ELISA (shown is the mean ± SD; ***p  <  0.001) (n = 3). **(C,D)** Wt BMDCs were treated with Propranolol (1 µM) and stimulated with Noradrenalin (10 µM) and IL-33 (100 ng/ml). Supernatant were collected and analyzed by ELISA (shown is the mean ± SD; ***p  <  0.001) (n = 4). **(E)** Wt BMDCs were stimulated with either Noradrenalin (10 µM) or IL-33 (100 ng/ml) or both together. Lysates were analyzed by Western blotting. The unstimulated control was set as 1 (shown is the mean ± SD of n = 3 independent experiments; *p <  0.05, **p  <  0.005 and ***p  <  0.001). The original blots are shown in Supplementary Fig. 8.

Adrenergic receptors are negative regulators of TIR family member-mediated signaling^23,33^. We investigated, whether stimulation of adrenergic receptors influence the IL-33-induced cytokine response in BMDCs. As shown in Fig. 4A,B treatment with Noradrenalin strongly reduced the IL-33-induced production of TNFα, but did not affect the IL-6 production. Thereby, 1 µM and 10 µM Noradrenalin are equally effective to reduce the IL-33-induced TNFα response **(**Fig. 4A**)**. Next, we tested the stimulation sequence with Noradrenalin and IL-33. Simultaneous or pre-incubation with Noradrenalin for 30 min most efficiently blocked the IL-33-induced TNFα production **(**Fig. 4C**)**. Stimulation of adrenergic receptors mediates the activation of adenylate cyclases and thereby the production of the second messenger cAMP^34,35^. Forskolin, an activator of the adenylate cyclases, strongly increases the production of cAMP^34^. Treatment of BMDCs with Forskolin blocked the IL-33-induced production of TNFα **(**Fig. 5A**)**, but not the IL-6 production **(**Fig. 5B**)**. This indicates that adrenergic receptors via cAMP inhibit IL-33-induced signaling pathways. Noradrenalin is a non-selective agonist of adrenergic receptors. Treatment of BMDCs with Propranolol, an antagonist of β-adrenergic receptors, reverses the effects of Noradrenalin on the IL-33-induced TNFα production whereas the production of IL-6 was not altered **(**Fig. 5C,D**)** indicating that Noradrenalin via β-adrenergic receptors controls the IL-33-induced TNFα production.

### Noradrenalin blocks the IL-33-induced activation of p38 and of JNK

Noradrenalin blocked the IL-33-induced production of TNFα but not of IL-6 in BMDCs, most likely by blocking essential signaling pathways involved in the IL-33-induced TNFα, but not IL-6 production. The production of TNFα but not of IL-6 depends on the p38-MK2/3 signaling module, and on JNK1/2. Thus we tested which of these signaling pathways are influenced by stimulation with noradrenalin. While Noradrenalin alone did not induce the activation of JNK1/2 and of p38 in BMDCs, treatment of BMDCs with noradrenalin equally reduced the IL-33-induced activation of JNK1 and JNK2 as well as of p38 **(**Fig. 5E**)**. These data demonstrate that β-adrenergic receptors specifically regulate the production of TNFα by controlling the IL-33-induced activation of p38 and JNK1/2.

## Discussion

We recently showed that the IL-33-induced TNFα production in BMDCs depends on the MyD88-IKK2-NF-κB signaling pathway, as well as on the p38-MK2/3 signaling module^5^. Here we show that JNK1/2 are also essential for the IL-33-induced TNFα production. Due to the involvement of JNK1/2 and the p38-MK2/3^5^ signaling module, we speculated that a crosstalk between these two MAPK pathways exists. However, IL-33 does not induce a crosstalk between JNK1/2 and p38 demonstrating that both MAPK pathways act independently to induce the production of cytokines in BMDCs. In contrast to IL-33, we assume that GM-CSF induces a crosstalk between the p38-MK2/3 signaling module and JNK1/2. Similar to mast cells^12,31,36^ the p38-MK2/3 signaling module limits the activation of JNK1/2 and thus the proliferation of BMDCs by feedback inhibition **(**Supplementary Fig. 3A**)**. However, the detailed mechanism behind this regulatory function is unknown. We speculate that MK2/3 generally limits the JNK1/2-dependent proliferation of innate cells by inducing the expression of MAPK phosphatases (MKPs)^37,38^.

Our data further indicate that the ligand-dependent mode of cooperation of different MAPK pathways mediates different cellular responses in DCs. Whereas the linked activation between the p38-MK2/3 signaling module and of JNK1/2 controls the GM-CSF-induced proliferation, the parallel activation of both MAPK pathways mediate the IL-33-induced cytokine production. Thereby the functional cooperation of JNK1/2 and the p38-MK2/3 signaling module together with NF-κB is essential for TNFα production in IL-33-activated BMDCs **(**Supplementary Fig. 3B–D**)**. In contrast to this, the IL-33-induced production of IL-6 neither depends on the p38-MK2/3 module nor on JNK1/2, but on NF-κB^5^ **(**Supplementary Fig. 3B–D**)**, whereas the production of IL-13 in BMDCs depends on the p38-MK2/3 signaling module, but not on NF-κB^5^. This underpins the essential and central role of JNK1/2 for the IL-33-induced TNFα production in BMDCs. However, there is no preference for a JNK isoform which mediates the IL-33-induced TNFα production. Neither inactivation of JNK1, nor of JNK2, influenced the IL-33-induced TNFα production. Only the pharmacological JNK inhibition by SP600125 strongly reduced the TNFα production induced by IL-33. This is explained by the fact that in contrast to a pan JNK inhibitor, in *jnk1*^−/−^ BMDCs the activation of JNK2L, and in *jnk2*^−/−^ BMDCs the activation of JNK1S are still intact. Therefore, the remaining JNK isoform together with the p38-MK2/3 signaling module and NF-κB is sufficient to mediate the IL-33-induced TNFα production. We hypothesize that JNK1/2 cooperatively with NF-κB mediate the transcription of TNFα. In contrast to this, the p38-MK2/3 signaling pathway stabilizes the TNFα transcripts **(**Supplementary Fig. 3B–D**)**^39^ and further mediates the translation of the TNFα transcripts via the mTOR-RSK pathway^5^.

The importance of JNK1/2 and p38 is further supported by the fact that both MAPK pathways and the resulting TNFα response are inhibited by activated β-adrenergic receptors **(**Supplementary Fig. 4A,B**)**. The mechanism underlying the inhibitory effect is unknown. However, β-adrenergic receptors activate the cAMP-dependent protein kinase A (PKA)-CREB signaling pathway^40,41^ which mediates the expression of MKPs and thus controls JNK1/2 and p38^37,38^. Therefore, we speculate that β-adrenergic receptors induce the expression of MKPs in BMDCs and thus limit the activation of p38 and JNK1/2 as well as the resulting TNFα production. In conclusion, by regulating JNK1/2 and p38, β-adrenergic receptors control the composition of IL-33-induced cytokine profiles of DCs **(**Supplementary Fig. 4A,B**)** and thus regulate their mediated effector functions. Given the fact that GM-CSF-generated BMDC resemble to inflammatory DCs^21^, the regulatory function of β-adrenergic receptors on IL-33-induced MAPK activation might also be important for DCs *in vivo*. Interestingly, an *in vivo* relevance of a crosstalk between the signaling of the IL-33R and β-adrenergic receptors has recently been shown in ILC-2. In these cells the IL-33-induced and p38-dependent IL-13 production^14^ is blocked by β_2_-adrenergic receptors and resulted in reduced inflammatory responses *in vivo*^42^. Together these data indicate that neuro-regulation of IL-33-induced effector functions on innate cells is a general mechanism to control and thus to avoid over-exuberant IL-33-induced inflammation. Therefore this provides novel therapeutic targeting strategies to modulate IL-33-induced inflammatory responses.

## Methods

### Mice

WT (C57BL/6 or Balb/c), Mapkapk2^tm1Mgl^ (*mk2*^−/−^) / Mapkapk3^tm1Mgl^ (*mk3*^−/−^)^39^, *myd88*^−/−^^43^, *jnk1*^−/−^^44^ and *jnk2*^−/−^^45^ mice were maintained at the Animal Research Facility of the Medical School, Hannover, Kiel and in the Animal Research Facility of the Jena University Hospital. We used sex- and age-matched knockout and wild type (wt) mice. Animals were housed according to the guidelines of the institutional and governmental committees for animal welfare. For this manuscript, we isolated organs from killed mice (mice strains see above). These organ isolations are approved by the appropriate governmental authority (Thüringer Landesamt für Lebensmittelsicherheit und Verbraucherschutz; Bad Langensalza).

### BMDC-generation

For generation of BMDCs we used the protocol as recently published^5^. In brief, bone marrow cells were seeded (2 × 10^5^ cells/ml) and after day 3, 6 and 8 medium [RPMI 1640 (Sigma Aldrich), with supplements and conditioned GM-CSF (20 ng/ml) supernatants from X63AG-GM-CSF cells] was refreshed. BMDCs were harvested (on day 9 or 10) and identified by surface expression of CD11c and CD11b (both from eBioscience) by flow cytometry.

### Flow cytometry

Staining was performed with antibodies in PBS (containing 0.25% BSA and 0.02% sodium azide) and propidium iodide (PI) (Biolegend) to exclude dead cells. We used anti-CD16/CD32 (clone 2.4G2) and rat-IgG (Jackson) to block non-specific binding. For identification of BMDCs we used anti-CD11b (PeCy7) (Biolegend) and anti-CD11c (APC) (Biolegend). For BMDC analysis we used a LSR II or Canto II flow cytometer (BD) and FlowJo version 9 (Tree Star, Inc., Ashland, OR) **(**Supplementary Fig. 1A**)**.

### Stimulation of BMDCs and lysis

Prior to stimulation, BMDCs were starved for GM-CSF for 1 h. Afterwards cells were pre-incubated for 30 min with inhibitors (as indicated in the Figures) (all Merck Millipore) and stimulated with IL-33 (Peprotech). In some experiments (as indicated in the Figures) BMDCs were treated with Noradrenalin (Sigma Aldrich) for 30 min and then stimulated with IL-33. Cell lyses was performed with a standard protocol^5^. Protein concentration was determined by using the BCA-assay (Pierce). Afterwards lysates were boiled in 6 × Laemmli buffer.

### Immunoblotting

Immunoblotting was performed with a standard protocol^5^. We used primary antibodies against pSIKK1/2/ IKK1/2, pT/Y-p38/ p38, anti-MK2, anti-MK3, pJNK1/2/ JNK1/2 and JNK1 (all Cell Signaling except anti-IKK1/2 and tubulin which were from Santa Cruz) and secondary antibodies conjugated with HRP [anti-rabbit-Ig, anti-goat-Ig (both Santa Cruz) and anti-mouse-Ig (Thermo Fisher Scientific)]. Detection was performed using ECL reagent (Pierce). Western blots were digitally developed with the ImageQuant 4000 system (GE Healthcare Life Science, England) or with X-Ray films (Fuji).

### ELISA

For ELISA experiments BMDCs were seeded (10^6^ cells/ml) in GM-CSF-free medium. BMDCs were pre-incubated with DMSO (vehicle), inhibitors (30 min) (all from Merck  Millipore) (concentrations are shown in the Figure legends)  or  Noradrenalin and Propranolol (both from Sigma Aldrich) . Afterwards BMDCs were stimulated with IL-33 (Peprotech). Supernatants were collected (after 24 h) and analyzed for TNFα and IL-6 by using matched pair antibodies (eBioscience).

### Proliferation assays

BMDCs were starved for GM-CSF (1 h). [3 H]-thymidine (1µCi) in 25 µl complete IMDM (PAA) (without GM-CSF) was added. Cells were incubated with [3 H]-thymidine (1µCi) for 18 h. Radioactivity was determined by using the β-scintillation counter (Perkin Elmer).

### Statistical analysis

For the generation of BMDCs, the bone marrow of several mice was pooled. For ELISA experiments, every biological replicate was split into at least a 6-fold determination (technical replicates). Western blots intensities were quantified with the Image J software (Fiji; Freeware). The phosphorylation blots were normalized to the total protein western blots. Afterwards the control (unstimulated sample) (if not stated otherwise) of wt BMDCs or of DMSO treated BMDCs was set as 1. The cytokine concentration is indicated as the mean of measurements ± standard deviation (SD). Statistical analysis was performed with Graph Pad Prism 5 by using the unpaired students *t*-test. Statistical significance was assessed for p ≤ 0,05 (*p ≤ 0.05; **p ≤ 0.01; ***p ≤ 0.001).

## Supplementary information

Supplementary information.
