# Supplementary material for: The IL-33-induced p38-/JNK1/2-TNFα axis is antagonized by activation of β-adrenergic-receptors in dendritic cells
Source: Sci Rep. 2020 May 18;10:8152. doi: 10.1038/s41598-020-65072-3 (PMC7235212; doi:10.1038/s41598-020-65072-3)

The IL-33-induced p38-/JNK1/2-TNFα axis is antagonized by activation of β-adrenergic-receptors in dendritic cells

Christiane Helbig, Franziska Weber, Nico Andreas, Thomas Herdegen, Matthias Gaestel, Thomas Kamradt and Sebastian Drube

Supplementary Figure Legends

Supplementary Figure 1. MyD88, NF-κB, p38 and MK2/3 mediate the IL-33-induced TNFα production in BMDCs.

(A) Shown is the gating strategy for identification of *in vitro* generated BMDCs (see Methods section). **(B)** Wt BMDCs were stimulated with IL-33 as indicated (n=3). **(C)** Wt and *myd88*$^{-/-}$ BMDCs were stimulated with IL-33 (100ng/ml) (n=3). **(D)** Wt BMDCs were pretreated with the NF-κB inhibitor (10µM) (NF-κBi). Afterwards cells were stimulated with IL-33 (100ng/ml) (n=3). **(E)** Wt BMDCs were pretreated with SB203580 (2.5µM) (p38i). Afterwards cells were stimulated with IL-33 (100ng/ml) (n=3). **(F)** Wt and *mk2*$^{-/-}$/*3*$^{-/-}$ BMDCs were stimulated with IL-33 (100ng/ml) (n=3). **(B-F)** Supernatants were collected and analyzed for TNFα. Shown is the mean ± SD; ***$p < 0.001$.

Supplementary Figure 2. JNKs are dispensable for the IL-33-induced activation of IKK2 and p38. (A, B) Wt BMDCs were treated with the JNK inhibitor SP600125 (10µM) and subsequently stimulated with IL-33 (100ng/ml). Lysates were analyzed by Western blotting. Blots of 3 independent experiments with BMDCs separately generated from wt mice were quantified, and statistically analyzed. The control of unstimulated wt BMDCs (DMSO) was set as 1 (shown is the mean ± SD; ns: not significant). **(C-F)** Wt, *jnk1*$^{-/-}$ **(C, D)** and *jnk2*$^{-/-}$ **(E, F)** BMDCs were stimulated with IL-33 (100ng/ml). Lysates were analyzed by Western blotting. Blots of 5 (for wt/ *jnk1*$^{-/-}$) and 4 (for wt/ *jnk2*$^{-/-}$) independent experiments with BMDCs separately generated from wt, *jnk1*$^{-/-}$ or *jnk2*$^{-/-}$ mice were quantified, and statistically analyzed. The control of the unstimulated wt BMDCs was set as 1 (shown is the mean ± SD; ns: not significant). **(G)** Wt, *jnk1*$^{-/-}$ and *jnk2*$^{-/-}$ were stimulated with IL-33. Supernatants were analyzed by ELISA for TNFα (n=4) and IL-6 (n=5). Shown is the

mean ± SD (**ns**: not significant). **(H)** Wt or *mk2*$^{-/-}$/*3*$^{-/-}$ BMDCs were treated with [^{3}H]-thymidine (1µCi). Samples were analyzed with a β-scintillation counter. Shown is cpm (counts per minute in wt compared to *mk2*$^{-/-}$/*3*$^{-/-}$ BMDCs). Shown is the mean ± SD; ***$p < 0.001$.

Supplementary Figure 3. Proposed models of the signaling pathways induced by GM-CSF and IL-33 in DCs. (A) GM-CSF (G) activates MK2/3 which controls the IKK-JNK1/2 signaling pathway and the proliferation. **(B)** IL-33 induces the activation of NF-κB, JNK1/2 and of the p38-MK2/3 signaling module. Whereas JNK1/2 contributes to the transcriptional TNFα response, the p38-MK2/3 signaling module mediates the stability of TNFα transcripts. In contrast to the TNFα production, the production of IL-6 is independent of the p38-MK2/3 signaling module. **(C)** Inhibition of JNK1/2 with SP600125 bocks the IL-33-induced TNFα production, whereas the production of IL-6 is not influenced. **(D)** Inactivation of the p38-MK2/3 signaling module destabilizes the TNFα transcripts resulting in the blockade of the TNFα production. In contrast to this, the production of IL-6 is not influenced.

Supplementary Figure 4. Proposed models for the regulation of the IL-33-induced signaling pathways by adrenergic receptors. (A) Noradrenalin blocks the IL-33-induced activation of JNK1/2 and p38. In contrast to the IL-33-induced IL-6 production, the production of TNFα depends on JNK1/2 and the p38-MK2/3 signaling module. Therefore, activation of β-adrenergic receptors selectively blocks the IL-33-induced TNFα, but not the IL-6 production. **(B)** Propranolol, a Noradrenalin antagonist blocks the binding of Noradrenalin to β-adrenergic receptors. Thus IL-33 induces the activation of JNK1/2 and the p38-MK2/3 signaling module and the production of TNFα.

Supplementary Figure 5. Original blots for Figure 1A and 2A. On the left hand side the unlabeled blots with two exposure times and photographs of the membranes with indicated molecular weights (MW) are shown. The longer exposure and the photographs of the membrane are shown to indicate the contours of the blots. On the right hand side the same blots are shown. In addition, on these blots we indicate the contours of the membranes by marked frames and the image which is shown in the appropriated figures. These Western blots were digitally developed with the ImageQuant 4000 system (GE Healthcare Life Science, England).

Supplementary Figure 6. Original blots for Figure 2B. See description of the **Supplementary Figure 5.**

Supplementary Figure 7. Original blots for Figure 3A. See description of the **Supplementary Figure 5.**

Supplementary Figure 8. Original blots for Figure 3B and 5E.

For **Figure 3B** see description of the **Supplementary Figure 5.**

The original blots for **Figure 5E** were developed with X-Ray films.

Supplementary Fig. 1

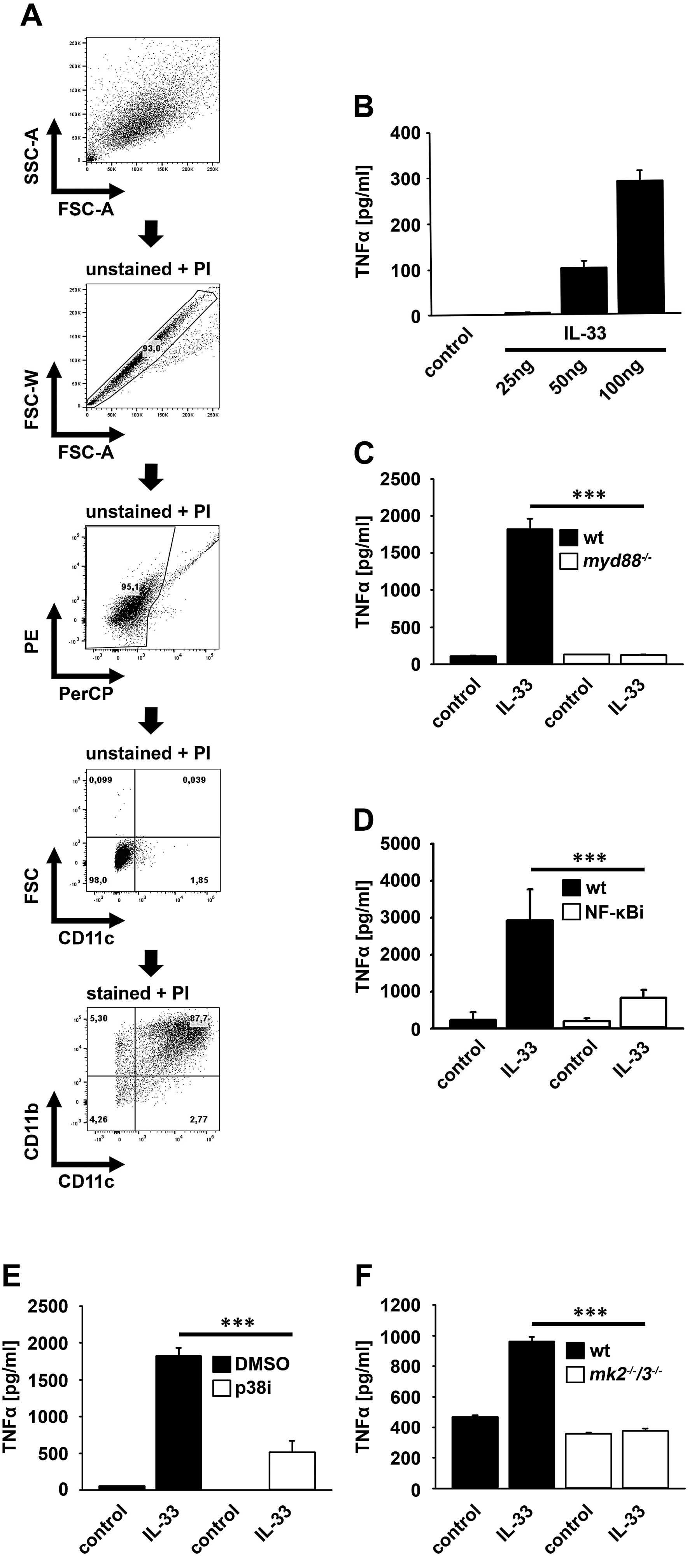

Supplementary Fig. 2

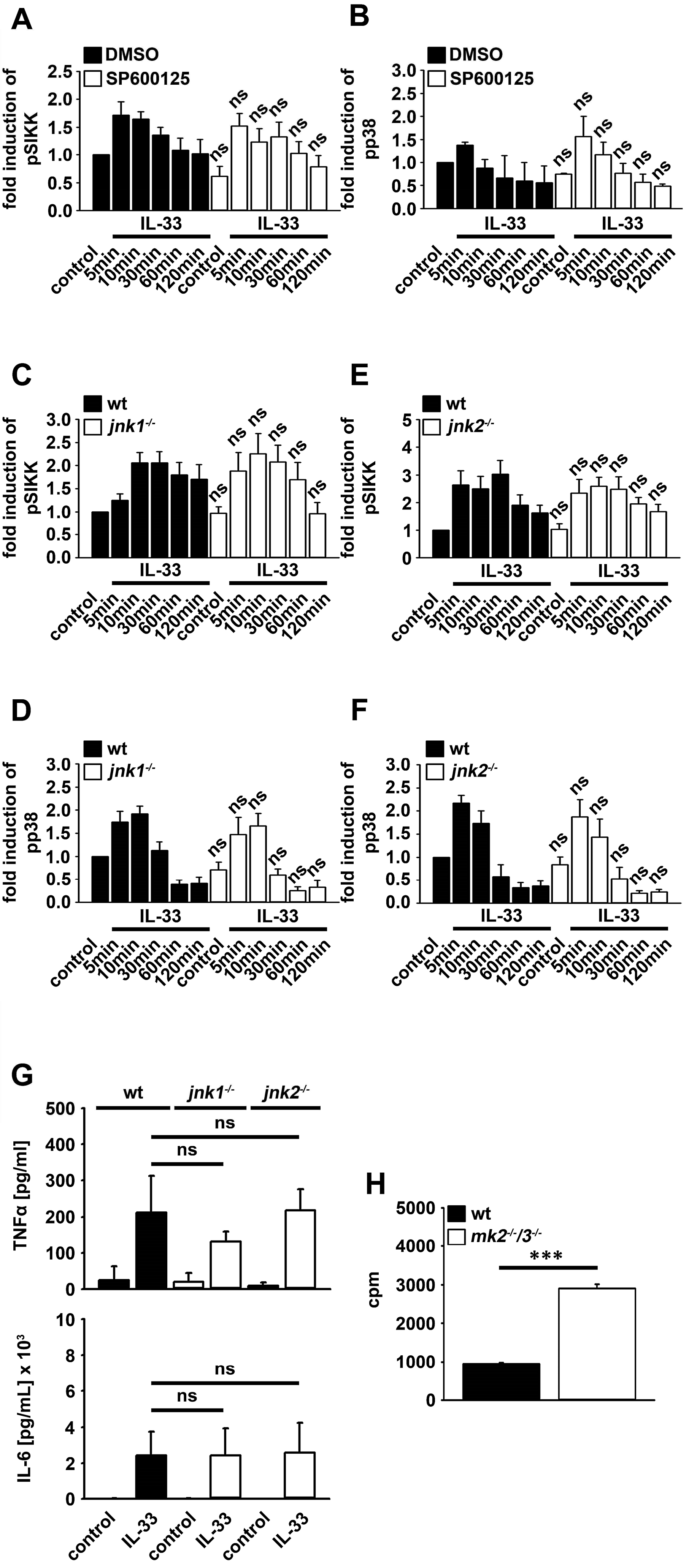

Supplementary Fig. 3

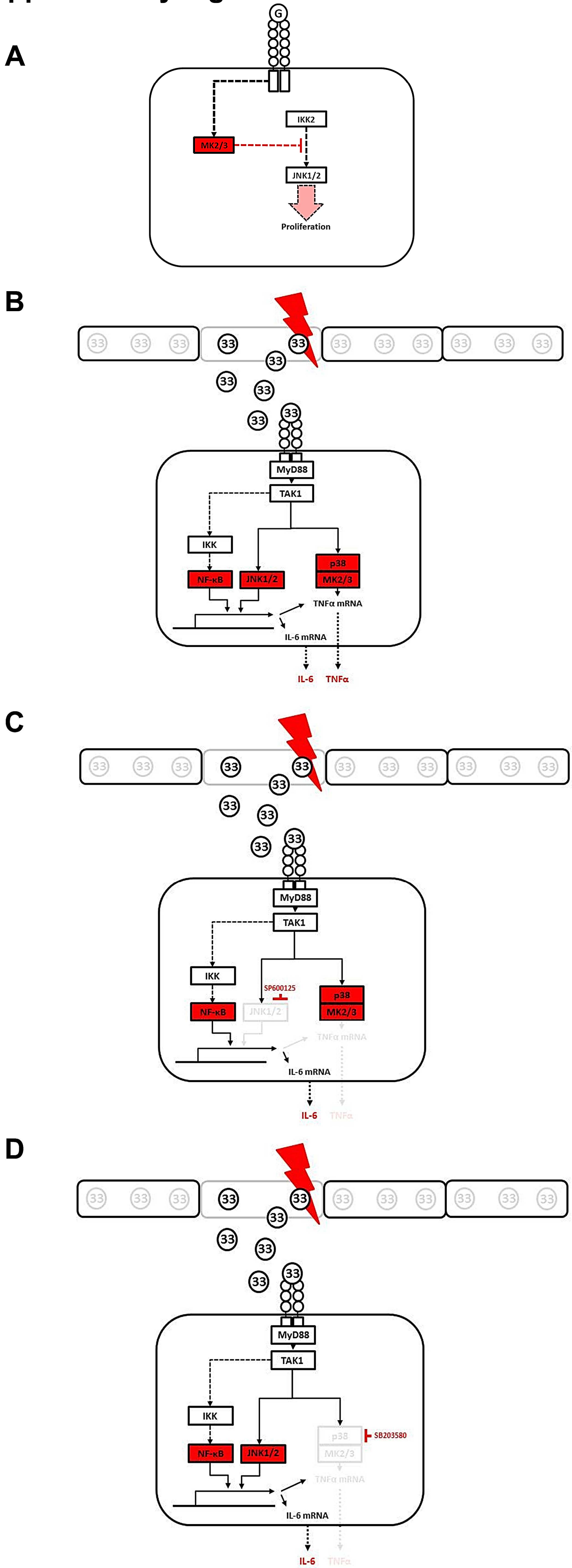

Supplementary Fig. 4

A

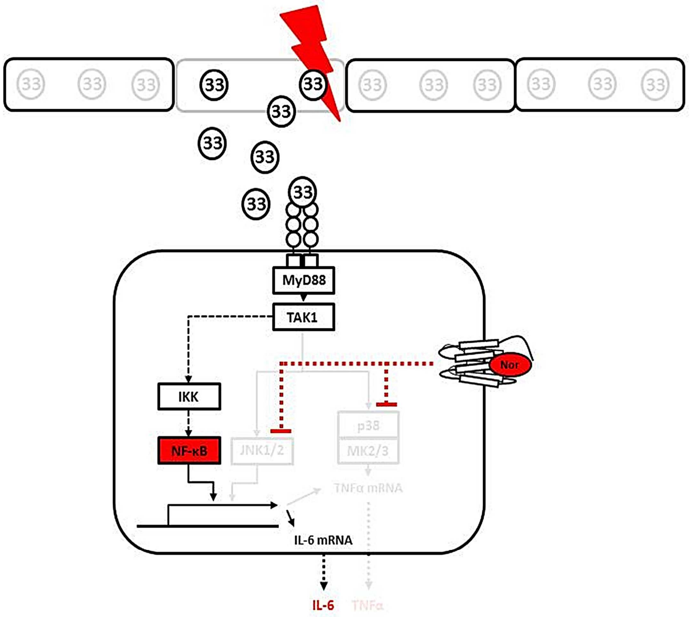

B

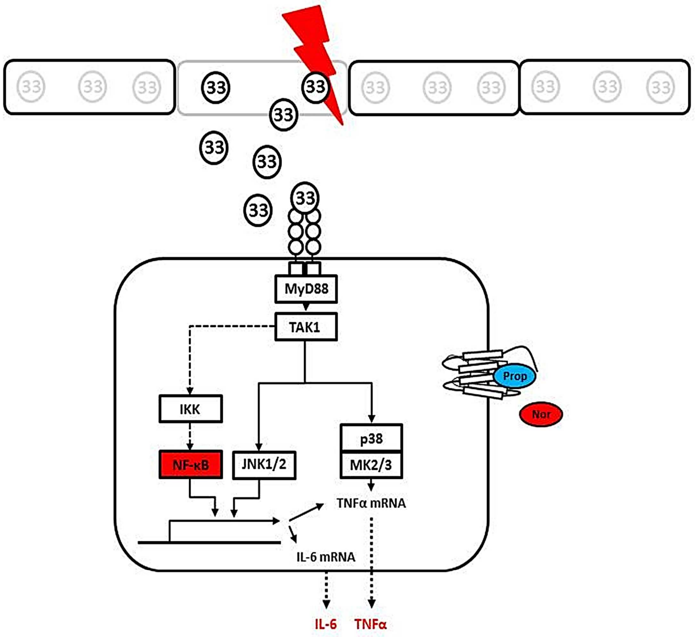

Supplementary Fig. 5 original westerblots

Fig. 1A

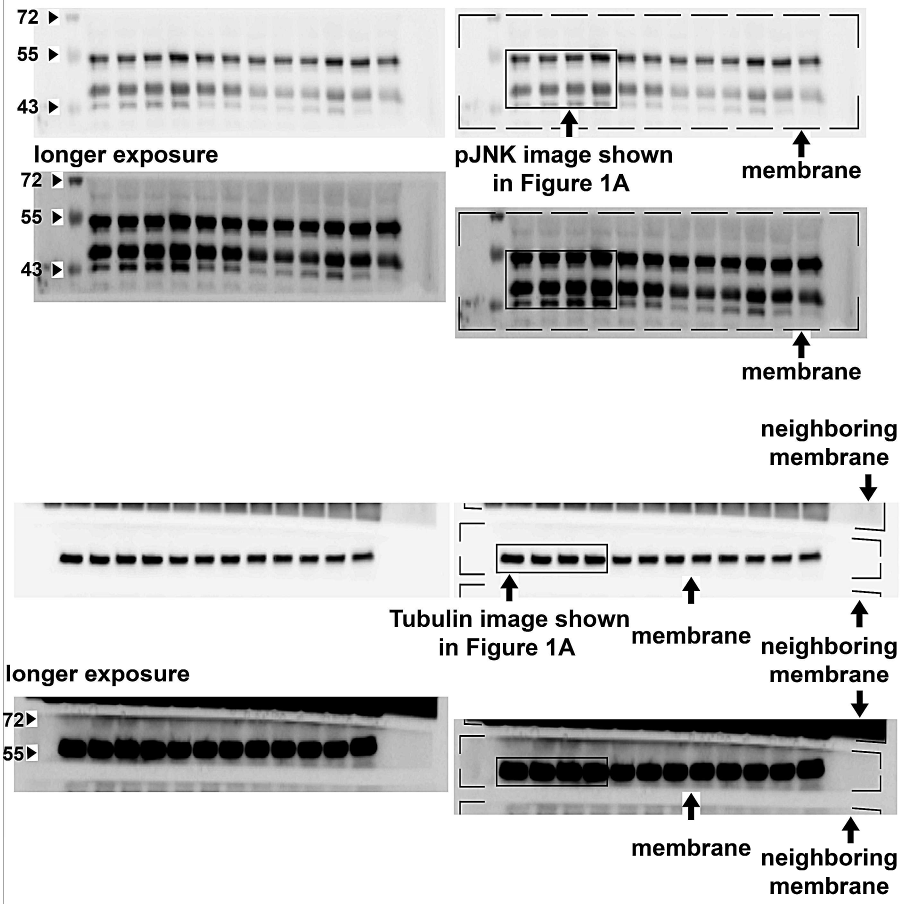

Fig. 2A

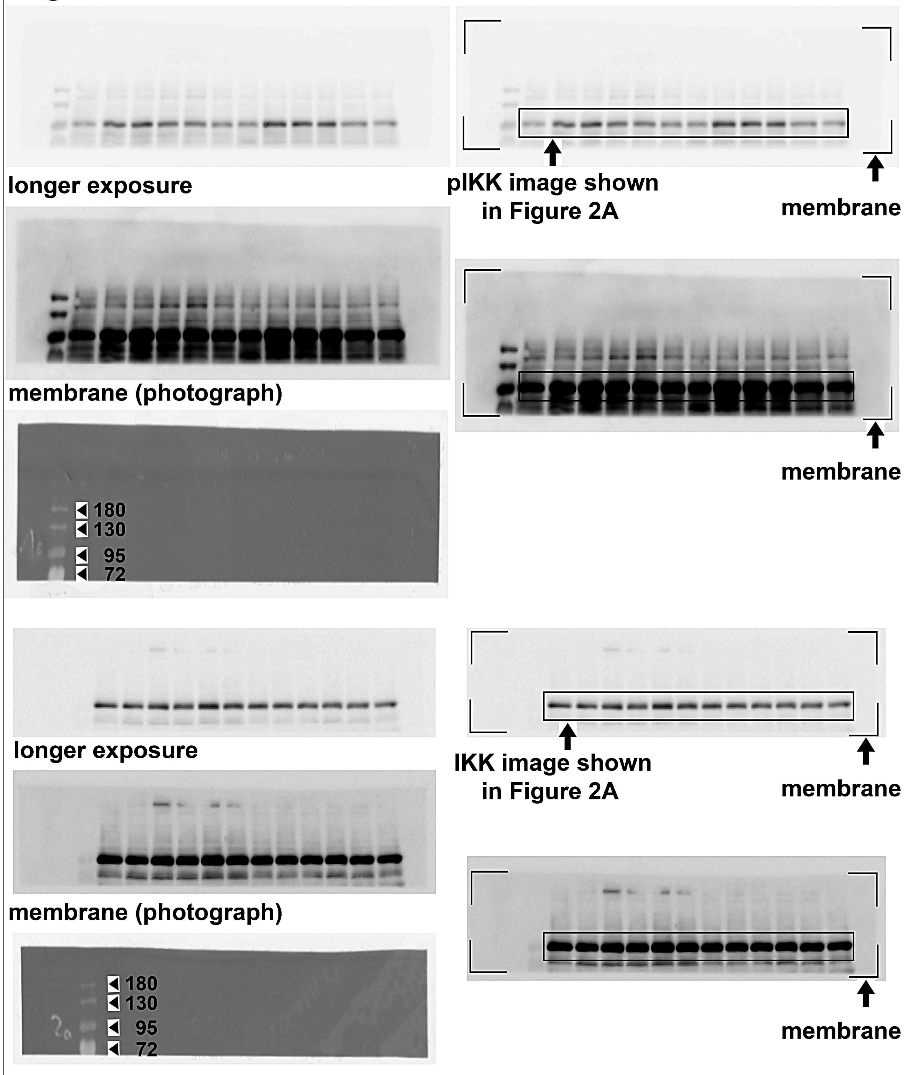

Supplementary Fig. 6 original westerblots
Fig. 2B

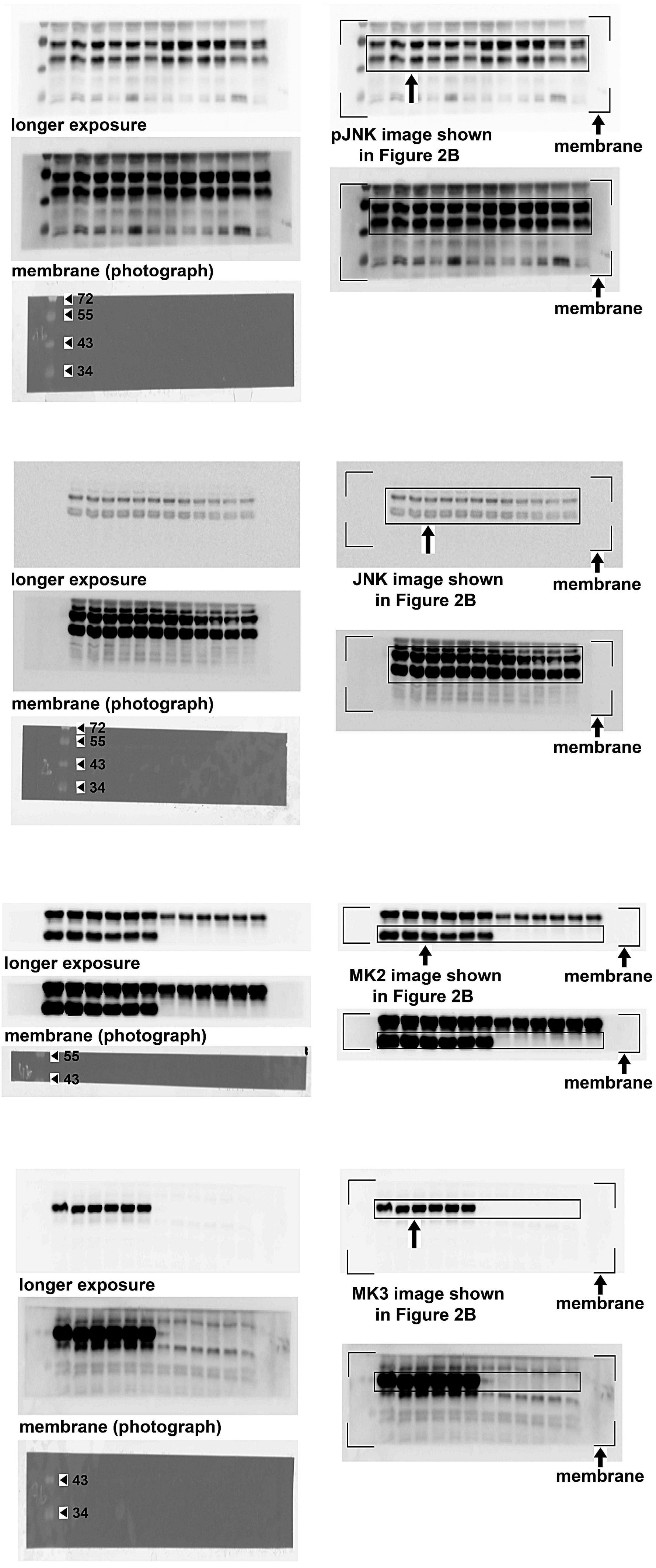

Supplementary Fig. 7 original westerblots

Fig. 3A

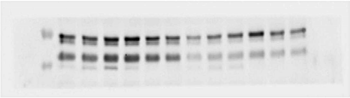

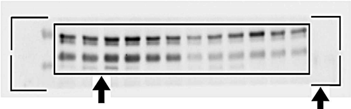

pJNK image shown in Figure 3A

membrane

longer exposure

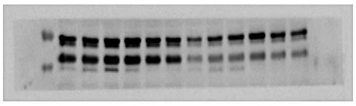

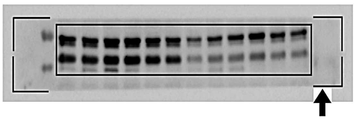

membrane

membrane (photograph)

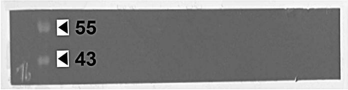

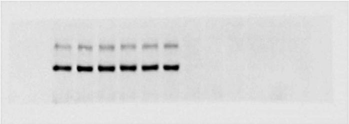

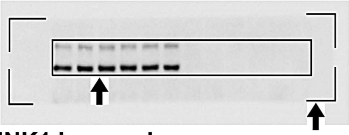

JNK1 image shown in Figure 3A

membrane

longer exposure

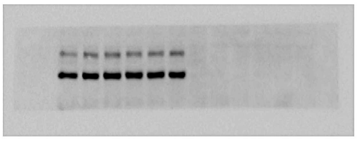

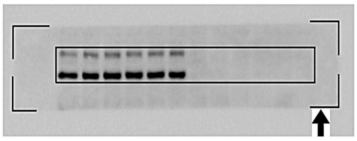

membrane

membrane (photograph)

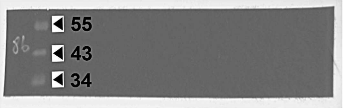

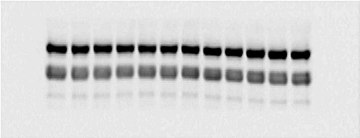

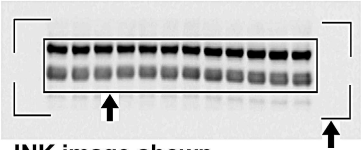

JNK image shown in Figure 3A

membrane

longer exposure

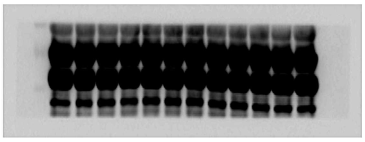

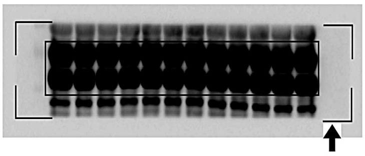

membrane

membrane (photograph)

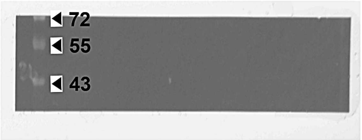

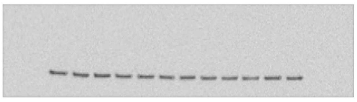

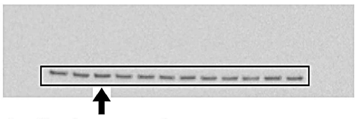

Tubulin image shown in Figure 3A

longer exposure

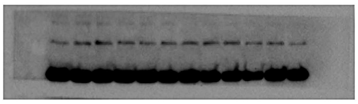

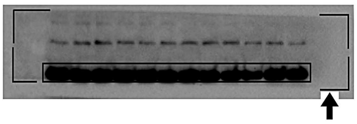

membrane

membrane (photograph)

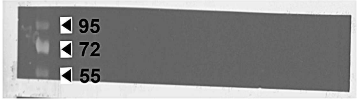

Supplementary Fig. 8 original westerblots

Fig. 3B

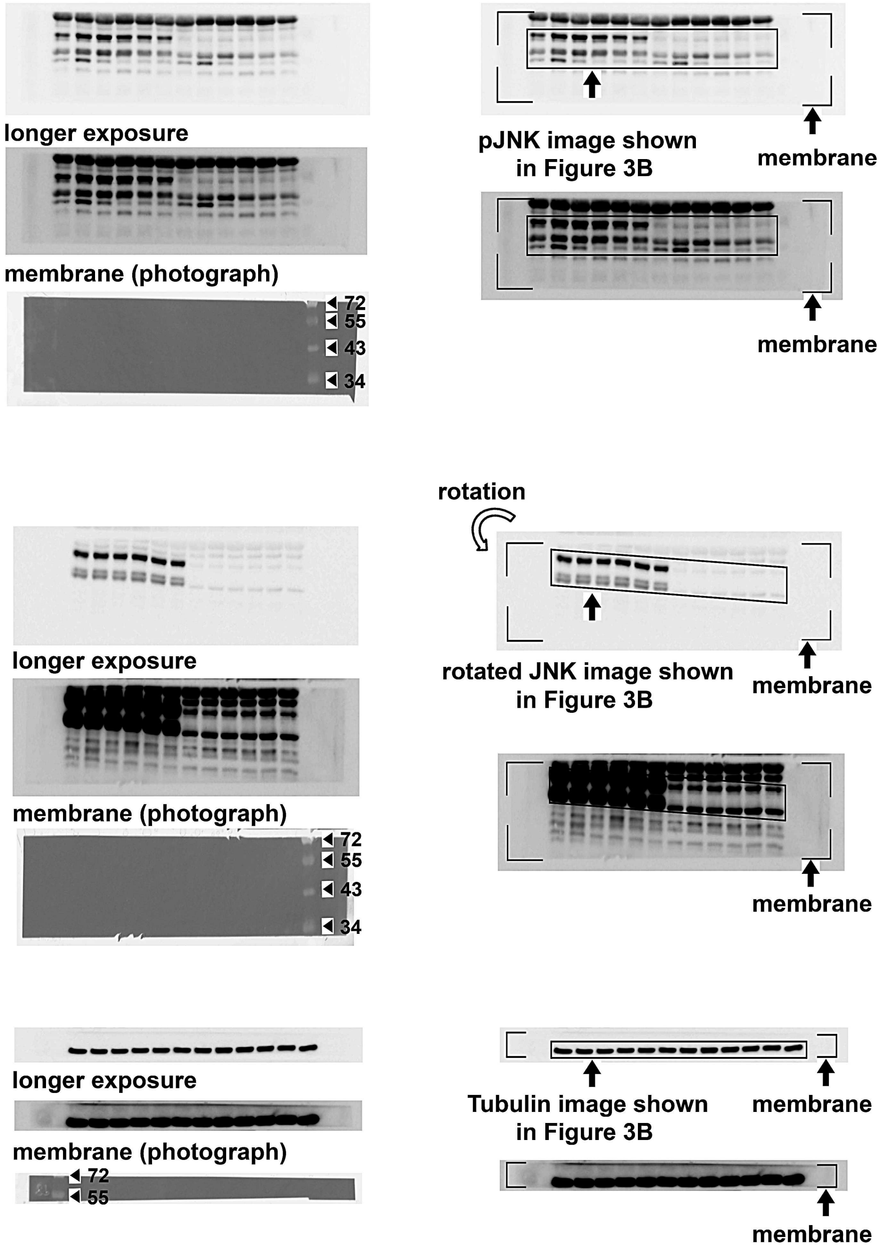

X-ray films of Figure 5E

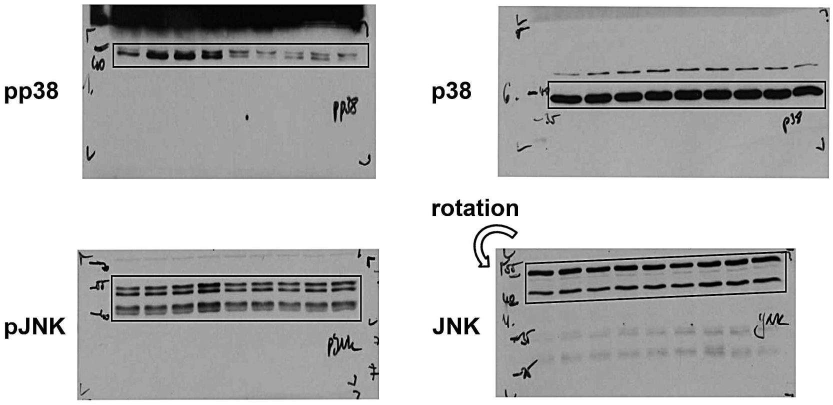

Supplement: Supplementary file 1 — Supplementary information. [file 41598_2020_65072_MOESM1_ESM.pdf]
